# Progress in Medicine

**Published:** 1896-10-31

**Authors:** 


### Progress in Medicine.

### DISEASES OF THE INTESTINES.

An interesting account of a very localised outbreak of dysentery is given by Finney (*Dublin Journ. Med. Science*, May, 1896). Five people out of a family of seven were attacked, and two died—a child of eleven months, and its grandmother, aged seventy. In all there was abdominal pain, tenesmus, and the frequent passage of small motions containing blood and mucus. No definite clue was to be found as to the cause of the outbreak. The family, however, had been supplied for two days with the milk from the cow of a friend, and, at the same time, the child of that friend died of intestinal disease. The question therefore suggested itself as to whether it was possible that infection had taken place through the milk. The house was part of business premises in which several clerks, as well as three or four domestics resided, but none of them were attacked.

Rolleston (*B. M. J.*, May 30th, 1896) and Haward record a case of that not very common condition, chronic dilatation of the colon. A boy at twelve had been subject to constipation since six months of age. On admission to St. George's Hospital he was emaciated, the eyes were sunken, and the abdomen was enormously distended, over which peristaltic movements of the intestines could be seen. The bowels acted sometimes fairly well, but he used to have recurring attacks of constipation and vomiting, and in one of these he died. At the post-mortem examination great dilatation of the colon was found. There was no stricture or obstruction to account for it. The muscular wall of the colon was considerably hypertrophied, and there were numerous ulcers, presumably due to distension.

Intestinal indigestion is the subject of a paper by Christopher (*Therapeutic Gazette*, March 16th, 1896). He mentions that the normal digestion of starches occurring under the influence of physiological amylolytic ferments leads to the formation of fermentable sugars, and that these sugars are usually transformed in the small intestine into various acids and gases by the action of various micro-organisms. At times this secondary bacterial fermentation occurs in excess, and constitutes the condition known as intestinal indigestion. Why this excessive decomposition of the sugars should occur is not clear, but Dr. Christopher thinks that the treatment indicated is starvation of the bacteria. This may be done in two ways. Either carbohydrate food may be withheld for a time, or the carbohydrates may be digested before they reach the intestines. In order to obtain the latter result he employs some diastasic ferment immediately after meals, which digests the starch before the secretion of hydrochloric acid has reached such a degree as to interfere with the diastasic action. Taka-diastase, given in doses of from three to five grains, he has found useful. Brunton (*Lancet*, May 30th, 1896) gives several interesting and useful points to be noticed in the treatment of constipation and diarrhœa. After mentioning the well-known facts that constipation may be due to lack of food that mechanically stimulates the intestines, to insufficient quantity of fluid, or to want of exercise, he states that constipation occasionally appears to be due not to an indolent condition of the intestines, but to be dependent upon reflex inhibition of the peristaltic movements. In these cases, which are not uncommon in delicate women suffering from some kind of ovarian or uterine disease, exercise instead of removing the constipation renders it most obstinate. Under these circumstances rest, and sometimes opium, will be found useful. When speaking of diarrhœa, Dr. Brunton refers to the form known as "morning diarrhœa." In this variety three to six movements may occur before eleven a.m., and then the patient is comfortable for the remainder of the day. Brunton considers that the best remedy is to take no liquid after five o'clock in the afternoon. He thinks that fluid taken after that time tends to accumulate in the stomach and intestines, instead of being absorbed. In some cases dilatation of the stomach is present, but apparently not in all. An interesting form of treatment for summer diarrhœa in children is given by Reinach (*B. M. J.*, July 11th, 1896). It is pointed out that rational treatment is directed to giving rest to the diseased tract, and to prevent the thickening of the blood secondary to the loss of fluid. This writer has treated fifteen cases of infantile diarrhœa with subcutaneous injections of sterile serum, since injections of serum apparently lead to thinning of the blood, and the treatment also provides a small amount of nourishment while food is withheld from the alimentary canal. Of the fifteen cases treated four died, but two of these had broncho-pneumonia. The value of tannalbin in diarrhœa forms the subject of a paper by Engel (*Med. Chronicle*, June, 1896; *Am. Surg. Bulletin*, March 21st, 1896). It is first pointed out that tannin would be a good intestinal astringent were it not for the fact that it combines with albuminous substances, and consequently exerts its astringent effects higher up, namely, in the stomach. A tannin albuminate, tannalbin, has been devised to obviate these results. This remains unaltered in the stomach, but when it meets with an alkali the tannin is set free. The drug was tested clinically in forty cases. In a few instances diarrhœa of from two to seven weeks' duration was arrested in one or two days by full doses. A few cases of failure were noted, but in these were severe anatomical lesions, such as tuberculous ulcers. The gastric functions were not interfered with in any way. Ogilvie (*Lancet*, June 20th, 1896) reports further cases of tapeworm treated by very large doses of male fern. Thus he gave one drachm of the liquid extract to a girl at seven years, and followed it up at the end of an hour by giving another dose of forty-five minims. Ordinary doses had been given previously with no success, but these large doses brought away the worm entire. An erythematous rash, however, appeared during the day, but the child seemed in no way affected, and ran about as usual.

